# Genetic Polymorphisms in Estrogen-Related Genes and the Risk of Breast Cancer among Han Chinese Women

**DOI:** 10.3390/ijms16024121

**Published:** 2015-02-13

**Authors:** Min-Ying Sun, Hong-Yan Du, An-Na Zhu, Hui-Ying Liang, Gorka Ruiz de Garibay, Fen-Xia Li, Ming Li, Xue-Xi Yang

**Affiliations:** 1 Guangzhou Center for Disease Control and Prevention, Guangzhou 510440, China; E-Mails: sunmy1220@163.com (M.-Y.S.); lianghuiying@hotmail.com (H.-Y.L.); 2 School of Biotechnology, Southern Medical University, Guangzhou 510515, China; E-Mails: gzduhongyan@126.com (H.-Y.D.); zhuanna0215@aliyun.com (A.-N.Z.); lifenxia123@gmail.com (F.-X.L.); 3 Catalan Institute of Oncology, Bellvitge Institute for Biomedical Research, Barcelona 08908, Spain; E-Mail: gorkargp@hotmail.com

**Keywords:** estrogen, polymorphisms, breast cancer

## Abstract

Exposure to high levels of estrogen is considered an important risk factor for susceptibility to breast cancer. Common polymorphisms in genes that affect estrogen levels may be associated with breast cancer risk, but no comprehensive study has been performed among Han Chinese women. In the present study, 32 single-nucleotide polymorphisms (SNPs) in estrogen-related genes were genotyped using the MassARRAY IPLEX platform in 1076 Han Chinese women. Genotypic and allelic frequencies were compared between case and control groups. Unconditional logistic regression was used to assess the effects of SNPs on breast cancer risk. Associations were also evaluated for breast cancer subtypes stratified by estrogen receptor (ER) and progesterone receptor (PR) status. Case-control analysis showed a significant relation between heterozygous genotypes of rs700519 and rs2069522 and breast cancer risk (*OR* = 0.723, 95% *CI* = 0.541–0.965, *p* = 0.028 and *OR* = 1.500, 95% *CI* = 1.078–2.087, *p* = 0.016, respectively). Subgroup comparisons revealed that rs2446405 and rs17268974 were related to ER status, and rs130021 was associated with PR status. Our findings suggest that rs700519 and rs2069522 are associated with susceptibility to breast cancer among the Han Chinese population and have a cumulative effect with three other identified SNPs. Further genetic and functional studies are needed to identify additional SNPs, and to elucidate the underlying molecular mechanisms.

## 1. Introduction

Breast cancer is one of the most common malignancies in women, and it causes serious health issues in women worldwide [1]. Although the incidence rate of breast cancer is currently low in China compared to Western countries, it is increasing rapidly. If current trends continue, the incidence rate in China will rise to approximately 85 per 100,000 woman-years by 2021 [2]. It is widely recognized that breast cancer development is the consequence of complex interactions between the genome, lifestyle, and environment. Many factors including family history, high mammographic density, postmenopausal hormone use, early age of menarche, late first pregnancy, breastfeeding for short periods, lower parity, and a longer interval between births have been confirmed to be associated with breast cancer susceptibility [3]. In addition, inheritance and hormones have also been suggested to be important risk factors [4,5].

The normal growth and function of breast epithelial cells are under hormonal control. Estrogen acts as a key driver for breast cancer because it can increase the frequency of mitotic activity, thereby stimulating cell divisions and increasing the potential of cells to accumulate mutations [6,7]. It is well established that high levels of estrogen or prolonged exposure is associated with breast cancer susceptibility [8]. A reanalysis of nine prospective studies demonstrated that there was a statistically significant increase in breast cancer risk with increasing concentrations of endogenous sex hormones in postmenopausal women [9]. Thus, the proper level of endogenous estrogen is crucial for female health. The biosynthesis and metabolism of estrogens, the main source of endogenous estrogens, is a complex process. A series of enzymes, such as cytochrome P450, family 17 (CYP17), CYP11A, CYP19, hydroxysteroid (17-β) dehydrogenase 1 (HSD17B1), CYP1A1, CYP1A2, CYP1B1, catechol-*O*-methyltransferase (COMT), as well as many other estrogen-signaling regulatory enzymes, is involved in the catabolism of cholesterol into estrogens and their subsequent degradation [10]. To exert their physiological effects, estrogens must first bind to estrogen receptors (ERs) and form complexes that subsequently bind to specific sequences in the promoters of estrogen-responsive genes [11]. Thus, all of these estrogen-related genes have been speculated to influence the synthesis or degradation of estrogens and are considered as candidate breast cancer susceptibility genes.

Single-nucleotide polymorphisms (SNPs) are the most frequent type of variation in the human genome, and are also valuable in tests of association with common diseases and pharmacogenetic traits [12,13]. Many studies have evaluated the association between SNPs in estrogen-related genes, such as *CYP17*, *CYP19*, *CYP1A1*, *CYP1B1*, *COMT*, *GSTP1*, and *ER*α/β, and the risk of breast cancer, especially in postmenopausal women [14,15,16,17,18,19,20,21]. Polymorphisms in these genes have been reported to affect the regulation, transcription, and activity of the enzymes, as well as subsequent exposure to endogenous estrogen and the development of breast cancer [14]. Although the effect of individual SNPs is usually small, multi-SNP association analysis can reveal cumulative risk effects and significantly relate to breast cancer [22]. Except for some reviews in which data from different reports and races were summarized and synthetically analyzed, most studies to date have focused on Western women [14,15]. Chinese populations have only been analyzed through studies with small sample sizes where few genes in the estrogen pathways were included. These conditions are too limited to explore and demonstrate special genes or SNPs of breast cancer genes in Han Chinese women comprehensively.

In a previous study, we genotyped six SNPs in the estrogen biosynthesis gene *CYP11A1*, and three variants (rs2279357, rs2959003, and rs2959008) were identified as associated with susceptibility to breast cancer [23]. In the present study, we explored the role of genetic polymorphisms in additional estrogen-related genes in breast cancer risk to systematically identify additional risk polymorphisms. We selected 32 SNPs, including 14 located in estrogen biosynthesis genes (*CYP17A1*, *HSD17B1*, *CYP19A1*, and *STS*), seven in metabolism genes (*CYP1A1*, *CYP1A2*, *CYP1B1*, *COMT*, and *GSTP1*), six in estrogen receptors (*ER*α and *ER*β), and five in estrogen-signaling regulatory enzymes (*CARM1*, *CREBBP*, *NQO1*, *SRD5A2*, and *SΜLT1E1*). The study was performed on Guangdong Han Chinese women.

## 2. Results

### 2.1. Subject Characteristics and Quality Control of SNPs

The baseline characteristics of the 1076 subjects (546 controls and 530 cases) are shown in Table 1. The mean ages at the time of recruitment of the patients and controls significantly differed (48.48 ± 10.18 and 44.59 ± 11.26 years, respectively, *p* < 0.05); therefore, statistical analysis based on case-control comparisons was adjusted for age. The most common tumor histopathological type was invasive ductal carcinoma (IDC), accounting for 83.96% (445) compared to 4.91% (26) of other tumor types including invasive lobular carcinoma (ILC), mucin-producing carcinomas (Muc), and medullary carcinoma (Medul) and 11.13% (59) of unknown types. Only five patients (0.94%) presented with stage 0 (carcinoma *in situ*), and stages 1–4 accounted for 23.77%, 24.34%, 30.94%, and 8.87% of patients, respectively. The cases consisted of 270 (50.94%) ER-positive tumors, 190 (35.85%) ER-negative tumors, 237 (44.72%) PR-positive tumors, and 223 (42.08%) PR-negative tumors, whereas the remaining 70 tumors (13.21%) were untested.

Quality control of all of the SNPs, consisting of a Hardy-Weinberg equilibrium (HWE) *p* > 0.05 in healthy controls, a genotyping rate ≥ 85%, and a minor allele frequency (MAF) > 0.01 was conducted. Five SNPs (rs2830, rs4680, rs707762, rs17268974, and rs1695) that deviated from HWE were omitted from further case-control analysis.

### 2.2. Polymorphisms in Estrogen Biosynthesis Genes and Breast Cancer

We compared the genotypic and allelic frequencies of 11 SNPs in estrogen biosynthesis genes based on the case-control study and observed that rs700519 in *CYP19A1* was significantly associated with breast cancer risk. The frequency of the heterozygous genotype C/T was lower in the breast cancer group than in the control group (20.9% *vs.* 26.2%). Women who carried the C/T genotype and T allele could be protected against breast cancer (*OR* = 0.723, 95% *CI* = 0.541–0.965, *p* = 0.028 and *OR* = 0.772, 95% *CI* = 0.600–0.993, *p* = 0.044, respectively). The results are shown in Table 2. No significant association was identified for the other SNPs.

**Table 1:** Baseline characteristics of the study subjects.

Variable	Control (*n* = 546)	Case (*n* = 530)
Ethnicity	Han Chinese women	Han Chinese women
Residence	Guangdong, China	Guangdong, China
Mean age ± SD (years) *	44.59 ± 11.26	48.48 ± 10.18
Mean age of menarche ± SD (years)	14.50 ± 1.54	14.33 ± 1.62
Mean BMI ± SD (kg/m^2^)	23.33 ± 3.58	23.57 ± 3.34
Exogenous hormone use ^a^ No	411 (75.27%)	373 (70.38%)
Yes	107 (19.60%)	114 (21.51%)
Unknown	28 (5.13%)	43 (8.11%)
No. full-term pregnancies Nulliparous	25 (4.58%)	37 (6.98%)
1	211 (38.64%)	214 (40.38%)
2	184 (33.70%)	172 (32.45%)
3	83 (15.20%)	66 (12.45%)
≥4	43 (7.88%)	41 (7.74%)
**Variable in breast cancer cases**		
Family history Yes	7 (1.32%)	
No	523 (98.68%)	
Tumor histopathology IDC	445 (83.96%)	
Others ^b^	26 (4.91%)	
Unknown	59 (11.13%)	
Clinical stage (UICC) ^c^ Stage 0 (*in situ*)	5 (0.94%)	
Stage 1	126 (23.77%)	
Stage 2	129 (24.34%)	
Stage 3	164 (30.94%)	
Stage 4	47 (8.87%)	
Unknown	59 (11.13%)	
ER status Positive	270 (50.94%)	
Negative	190 (35.85%)	
Unknown	70 (13.21%)	
PR status Positive	237 (44.72%)	
Negative	223 (42.08%)	
Unknown	70 (13.21%)	

Abbreviation: BMI, body mass index; * *p <*0.05; ^a^ Include either oral contraceptives (OC) or hormone replacement therapy (HRT); ^b^ Mean ILC, Muc and Medul; ^c^ mean International Union Against Cancer (UICC) stages; invasive ductal carcinoma (IDC); invasive lobular carcinoma (ILC); mucin-producing carcinomas (Muc); and medullary carcinoma (Medul); ER, estrogen receptor; PR, progesterone receptor.

**Table 2:** Single single-nucleotide polymorphisms (SNPs) in estrogen biosynthesis genes and association with breast cancer.

Gene	SNPs	Alleles	Codominant Model				*p*_trend_	Additive Model	
		Common/Rare	Heterozygote *OR* (95% *CI*) ^a^	*p* ^a^	Homozygote *OR* (95% *CI*) ^a^	*p* ^a^		Rare Allele *OR* (95% *CI*) ^a^	*p* ^a^
*CYP17A1*	rs743572	G/A	1.287 (0.978–1.694)	0.072	1.081 (0.756–1.547)	0.669	0.531	1.074 (0.902–1.278)	0.424
*HSD17B1*	rs615942	G/T	0.927 (0.694–1.237)	0.605	1.077 (0.760–1.526)	0.676	0.905	1.031 (0.866–1.227)	0.735
*CYP19A1*	rs2414096	G/A	1.099 (0.833–1.451)	0.504	1.133 (0.787–1.630)	0.503	0.441	1.065 (0.896–1.267)	0.457
*CYP19A1*	rs2445759	G/T	0.888 (0.359–2.193)	0.796	1.815 (0.153–21.572)	0.637	0.945	1.038 (0.471–2.288)	0.926
*CYP19A1*	rs2446405	T/A	0.957 (0.712–1.286)	0.769	0.830 (0.579–1.188)	0.307	0.458	0.919 (0.774–1.091)	0.336
*CYP19A1*	rs700519	C/T	**0.723 (0.541–0.965)**	**0.028**	0.845 (0.342–2.085)	0.715	**0.047**	**0.772 (0.600–0.993)**	**0.044**
*CYP19A1*	rs10046	T/C	0.785 (0.587–1.051)	0.103	0.748 (0.527–1.064)	0.106	0.074	0.866 (0.729–1.028)	0.100
*CYP19A1*	rs749292	G/A	0.994 (0.746–1.326)	0.970	1.100 (0.777–1.557)	0.593	0.627	1.045 (0.880–1.241)	0.618
*CYP19A1*	rs936306	G/A	0.869 (0.672–1.125)	0.287	0.869 (0.574–1.317)	0.508	0.386	0.908 (0.755–1.092)	0.305
*CYP19A1*	rs1008805	T/C	0.990 (0.766–1.278)	0.936	1.138 (0.736–1.758)	0.561	0.799	1.036 (0.860–1.248)	0.712
*CYP19A1*	rs11575899	TCT/DEL	1.029 (0.797–1.328)	0.826	0.954 (0.612–1.487)	0.837	0.883	1.001 (0.830–1.207)	0.992

Abbreviation: *OR*, Odds Ratio; 95% *CI*, 95% confidence interval; Significant values are marked in bold; ^a^ Adjusted by age.

### 2.3. Polymorphisms in Estrogen Metabolism Genes and Breast Cancer

Among the five SNPs in estrogen metabolism genes, the genotype distribution of *CYP1A2* rs2069522 was significantly different between cases and controls. In contrast to rs700519, the heterozygote T/C of rs2069522 increased the risk of breast cancer (*OR* = 1.500, 95% *CI* = 1.078–2.087, *p* = 0.016, Table 3). The other four SNPs showed no evidence of an association with breast cancer.

**Table 3:** Single SNPs in estrogen metabolism genes and association with breast cancer.

Gene	SNPs	Alleles	Codominant Model				*p*_trend_	Additive Model	
		Common/Rare	Heterozygote *OR* (95% *CI*) ^a^	*p* ^a^	Homozygote *OR* (95% *CI*) ^a^	*p* ^a^		Rare Allele *OR* (95% *CI*) ^a^	*p* ^a^
*CYP1A1*	rs1531163	A/G	0.996 (0.762–1.303)	0.978	0.643 (0.283–1.459)	0.290	0.618	0.938 (0.746–1.180)	0.586
*CYP1A1*	rs2606345	C/A	0.722 (0.479–1.088)	0.120	0.380 (0.034–4.241)	0.432	0.062	0.709 (0.480–1.048)	0.085
*CYP1A2*	rs2069522	T/C	**1.500 (1.078–2.087)**	**0.016**	0.514 (0.127–2.081)	0.351	0.091	1.305 (0.968–1.760)	0.081
*CYP1A2*	rs762551	A/C	0.876 (0.678–1.133)	0.313	0.873 (0.572–1.333)	0.529	0.362	0.914 (0.761–1.099)	0.339
*CYP1B1*	rs1056836	C/G	1.073 (0.787–1.462)	0.657	0.715 (0.286–1.792)	0.475	0.964	0.997 (0.761–1.306)	0.985

Abbreviation: *OR*, Odds Ratio; 95% *CI*, 95% confidence interval. Significant values are marked in bold. ^a^ Adjusted by age.

### 2.4. Polymorphisms in Estrogen Receptors (ERs) and Regulatory Genes

No significant differences were found in genotypic or allelic distributions between the case and control groups, indicating that the 11 SNPs in the *ERs* and regulatory genes were not independently associated with breast cancer in our study population. Detailed information is shown in Table 4 and Table 5.

**Table 4:** Single SNPs in estrogen receptors and association with breast cancer.

Gene	SNPs	Alleles	Codominant Model				*p*_trend_	Additive Model	
		Common/Rare	Heterozygote *OR* (95% *CI*) ^a^	*p* ^a^	Homozygote *OR* (95% *CI*) ^a^	*p* ^a^		Rare Allele *OR* (95% *CI*) ^a^	*p* ^a^
*ER*α	rs728524	A/G	1.137 (0.873–1.480)	0.340	0.628 (0.318–1.239)	0.180	0.965	0.996 (0.801–1.238)	0.972
*ER*α	rs2234693	T/C	1.110 (0.839–1.469)	0.465	0.986 (0.663–1.465)	0.944	0.892	1.022 (0.844–1.237)	0.823
*ER*α	rs9340799	A/G	0.945 (0.726–1.229)	0.671	1.222 (0.677–2.207)	0.505	0.900	1.011 (0.818–1.250)	0.918
*ER*α	rs3798577	T/C	0.796 (0.605–1.048)	0.104	0.927 (0.654–1.314)	0.669	0.378	0.936 (0.786–1.113)	0.454
*ER*β	rs1255998	G/C	0.929 (0.712–1.212)	0.585	0.979 (0.683–1.404)	0.908	0.783	0.976 (0.818–1.164)	0.783
*ER*β	rs1256049	G/A	0.951 (0.725–1.247)	0.716	1.075 (0.753–1.533)	0.692	0.925	1.022 (0.858–1.217)	0.805

Abbreviation: *OR*, Odds Ratio; 95% *CI*, 95% confidence interval; ^a^ Adjusted by age.

**Table 5:** Single SNPs in estrogen-signaling regulatory enzymes and association with breast cancer.

Gene	SNPs	Alleles	Codominant Model				*p*_trend_	Additive Model	
		Common/Rare	Heterozygote *OR* (95% *CI*) ^a^	*p* ^a^	Homozygote *OR* (95% *CI*) ^a^	*p* ^a^		Rare Allele *OR* (95% *CI*) ^a^	*p* ^a^
*CARM1*	rs12460421	G/A	1.272 (0.947–1.708)	0.110	1.024 (0.720–1.455)	0.896	0.980	1.018 (0.857–1.209)	0.841
*CREBBP*	rs130021	A/G	1.150 (0.877–1.507)	0.312	0.994 (0.691–1.431)	0.976	0.930	1.024 (0.859–1.220)	0.792
*NQO1*	rs1469908	G/A	1.142 (0.876–1.490)	0.326	0.889 (0.435–1.819)	0.748	0.380	1.069 (0.856–1.335)	0.555
*SRD5A2*	rs523349	G/C	1.068 (0.810–1.409)	0.639	1.024 (0.719–1.458)	0.897	0.742	1.019 (0.857–1.212)	0.833
*SΜLT1E1*	rs3736599	G/A	0.983 (0.761–1.270)	0.896	1.105 (0.717–1.703)	0.652	0.668	1.024 (0.851–1.233)	0.801

Abbreviation: *OR*, Odds Ratio; 95% *CI*, 95% confidence interval; ^a^ Adjusted by age.

### 2.5. Cumulative Risk Analysis

*CYP11A1* encodes the key enzyme that catalyzes the initial and rate-limiting steps of steroid hormone synthesis. In our previous study, three SNPs, rs2279357, rs2959003, and rs2959008, in *CYP11A1* were validated to be associated with breast cancer risk (*OR* = 1.558, 95% *CI* = 1.092–2.222, *p* = 0.014; *OR* = 0.668, 95% *CI* = 0.460–0.971, *p* = 0.034 and *OR* = 0.669, 95% *CI* = 0.452–0.991, *p* = 0.045) [23]. To investigate whether these significant risk polymorphisms had a cumulative effect, an integrated analysis including these five SNPs was performed to evaluate the cumulative risk for breast cancer (Table 6). We defined the genotypes C/C of rs700519, T/C of rs2069522, and T/T of rs2279357, rs2959003, and rs2959008 as risk genotypes to conduct statistical analysis. Consistent with our hypothesis, women who carried more risk genotypes had a higher susceptibility for developing breast cancer (*p*_trend_ = 0.030). Women with five high-risk genotypes had a statistically significant 1.286 times higher risk of developing breast cancer than controls (*OR* = 2.286, 95% *CI* = 1.187–4.399, *p* = 0.013).

The admixture maximum likelihood (AML) method was employed to evaluate the cumulative effect from multiple variants. As expected, four SNPs (rs2279357, rs2959008, rs2959003, and rs2069522) were significant at the 5% level (*p* = 0.02, 0.02, 0.03, and 0.04) after adjusting for age based on the trend test for association. The other SNP (rs700519) failed to reach this threshold (*p* = 0.08).

**Table 6:** Cumulative risk of five susceptibility SNPs for breast cancer.

The Number of Risk Genotype	Control	Case	*OR* (95% *CI*) ^a^	*p* ^a^
1/5	220 (46.8%)	201 (41.4%)	1	-
2/5	99 (21.1%)	110 (22.6%)	1.223 (0.873–1.714)	0.242
3/5	81 (17.2%)	79 (16.3%)	1.118 (0.772–1.619)	0.556
4/5	55 (11.7%)	65 (13.4%)	1.327 (0.877–2.007)	0.181
5/5	15 (3.2%)	31 (6.4%)	**2.286 (1.187–4.399)**	**0.013**

The five SNPs include rs700519, rs2069522, rs2279357, rs2959003, and rs2959008. Significant values are marked in bold; ^a^ Adjusted by age.

The global test yielded a marginally significant association for the whole estrogen-related pathway (*p*_global_ = 0.047). Dividing into four functional sub-pathways for the global test revealed strong associations with the estrogen biosynthesis (*p*_global_ = 0.026) and metabolism (*p*_global_ = 0.014) sub-pathways. However, the other two sub-pathways showed no association with breast cancer.

### 2.6. Stratified Analysis Based on ER and Progesterone Receptor (PR) Status

Given the significance of hormone receptors in breast cancer progression and clinical diagnosis, stratified analysis, based on ER and PR status and genetic polymorphisms, was performed. Finally, three SNPs (rs2446405, rs17268974, and rs130021) were found to be distributed differently among breast cancer subgroups. Genotype A/A and allele A of rs2446405 showed a stronger association with ER(+) tumors than ER(−) (*OR* = 2.072, 95% *CI* = 1.147–3.742, *p* = 0.016 and *OR* = 1.341, 95% *CI* = 1.030–1.746, *p* = 0.030); however, for rs17268974, A/A was associated with ER(−) tumors (*OR* = 0.468, 95% *CI* = 0.243–0.900, *p* = 0.023). A/G carriers of rs130021 also showed a tendency toward PR(+) tumors (*OR* = 1.756, 95% *CI* = 1.164–2.648, *p* = 0.007). The complete results for the different inheritance models are shown in Table 7.

**Table 7:** Case-case comparisons for the associations of polymorphisms by subgroups of ER and PR status.

Subgroups	SNPs	Alleles	Codominant Model				*p*_trend_	Additive Model	
		Common/Rare	Heterozygote *OR* (95% *CI*)	*p*	Homozygote *OR* (95% *CI*)	*p*		Rare Allele *OR* (95% *CI*)	*p*
ER status	rs2446405	T/A	1.217 (0.779–1.900)	0.388	**2.072 (1.147–3.742)**	**0.016**	**0.019**	**1.341 (1.030–1.746)**	**0.030**
ER status	rs17268974	T/A	0.857 (0.564–1.303)	0.471	**0.468 (0.243–0.900)**	**0.023**	**0.043**	0.782 (0.598–1.022)	0.072
PR status	rs130021	A/G	**1.756 (1.164–2.648)**	**0.007**	1.188 (0.680–2.076)	0.545	0.205	1.193 (0.915–1.556)	0.192

Abbreviation: ER, estrogen receptor; PR, progesterone receptor; Significant values are marked in bold.

## 3. Discussion

In the present retrospective study, two SNPs, rs700519 and rs2069522, were identified as being associated with breast cancer risk. The non-synonymous coding SNP rs700519 (Arg264Cys) is located in exon 7 of *CYP19A1*, which encodes aromatase, the key enzyme in estrogen biosynthesis. Aromatase catalyzes the rate-limiting step in the conversion of testosterone and androstenedione to estradiol and estrone in a wide variety of tissues, including the ovary, placenta, brain, and adipose tissue [24]. The up-regulation of *CYP19A1* expression has been observed to contribute to breast cancer [25]. Individual genetic polymorphisms in this gene have also previously been reported to be associated with enzymatic activity (C1123T and rs28757184), with certain pathologic phenotypes including breast cancer (Trp39Arg), and with poor efficacy of cancer treatment (rs4646) [26,27,28,29,30]. Studies in Chinese women have identified that the common missense polymorphism rs700519 alone or combined with other polymorphisms could significantly modulate the risk of endometriosis [31], polycystic ovary syndrome [32], and survival of breast cancer [33]. In the present population, this SNP was significantly associated with susceptibility to breast cancer, consistent with a result based on North Indian women [18]. However, some studies showed no significant association [34,35,36]. Another key enzyme of the pathway is CYP1A2, which also plays a crucial role in estrogen metabolism. Genetic variants of *CYP1A2* have been shown to contribute to the risk of lung and breast cancer by interacting with environmental factors and drug metabolism through regulation of enzyme activity [37,38,39]. SNP rs2069522 is located at −2847 bp of *CYP1A2*, within a putative region that bidirectionally regulates the transcriptional activation of both *CYP1A1* and *CYP1A2* genes [40]. Despite the important function of rs2069522, its association with breast cancer risk has not been clearly demonstrated. Genetic studies have only shown that it had the potential to affect colorectal cancer risk and the treatment response to clozapine in schizophrenic patients among British Caucasians and Koreans, respectively [41,42]. However, those results were not significant after correction for multiple testing. Our study revealed for the first time that heterozygosity of rs2069522 significantly increased breast cancer risk. It is possible that this variant may influence the transcription and thus activity of CYP1A1 protein. It is particularly worth noting that the association of rs700519 and rs2069522 was only observed for heterozygotes, but not for homozygotes. One possibility is that the number of rare homozygotes for these two SNPs is too small to show significant evidence, which is consistent with the HapMap database and other reports [41,43]. Furthermore, the biological function of these two SNPs has not been clarified fully. Thus, further analyses based on a larger set of SNPs should be conducted to perform fine mapping of the locus, and to explore their biological functions and mechanisms in more detail.

Genetic polymorphisms in estrogen-related genes have been intensively investigated in many studies. However, these studies produced inconsistent results [15,16,17,18,21,31,44,45]. Several polymorphisms that were previously reported to contribute to breast cancer risk showed no significant association in our case-control analysis, including rs743572, rs1056836, rs1695, and rs2234693 [18,21,44,45]. The inconsistency might mainly be due to racial differences in susceptibility to breast cancer [46], because these studies were performed on various populations and subject numbers. For instance, variants in *CYP1B1* (rs1056836), *COMT* (rs4680), and *ER*α (rs9340799) were significantly related to breast cancer susceptibility among Caucasian women [20,21], but we failed to find the same associations in our study population. Thus, it is necessary to replicate these risk loci to validate specific variants for Han Chinese women.

Polymorphisms in genes could affect the levels of transcription, translation, and activity of enzymes and result in wide inter-individual variability of responses to carcinogenic substances. Although the effect of a single polymorphism on tumorigenesis is weak, multiple loci could combine to generate a cumulative risk that cooperates with environmental exposure [47]. We have previously investigated the polymorphisms in *CYP11A1* and found that rs2279357, rs2959003, and rs2959008 were related to breast cancer risk [23]. Combined with two associated variants in the present study, the cumulative analysis of these five verified variants revealed a much higher risk than a single SNP, a finding that agrees remarkably well with our expectation. The pathway-based AML global test also showed a significant association for the whole estrogen-related pathway that was much stronger for estrogen biosynthesis and metabolism sub-pathways. These findings support the multigenic nature of the etiology of breast cancer and need for gene–gene or locus–locus interactive studies on low-penetrance genes to comprehensively explore the underlying mechanisms of breast cancer.

It is necessary for all hormones to combine with their corresponding receptors to exert their biological functions. The expression of receptors, such as ER, PR, and human epidermal growth factor receptor-2 (HER2), is included in routine clinical classification and evaluation of breast cancer [48]. Their expression may also predict the response to targeted therapies such as tamoxifen and trastuzumab. Therefore, we also carried out stratified analyses based on ER and PR subtypes in the case group. Three SNPs (rs2446405, rs17268974, and rs130021) were confirmed to be associated with ER or PR status. Genetic changes in the receptors may influence their activity and therefore the risk and subtype of breast cancer.

Our study had some limitations. First, menopausal status can modify a woman’s risk of developing breast cancer, and the incidence rate differs between premenopausal and postmenopausal women. In the current study, we did not evaluate the associations for postmenopausal and premenopausal women separately because information on menopausal status was not obtained from most of the participants. Second, the statistical power for all of the 1076 subjects was larger than 83% to detect a log-additive genotype relative risk of 1.30 (Table S1). It was considered to be well powered to detect strong or moderate effect sizes of disease-predisposing variants, but smaller effects might have been missed. In fact, the exact powers of the two significant SNPs, rs700519 and rs2069522, were 68.12% and 83.69%, respectively. Furthermore, some variants in estrogen-related genes, such as *CYP1A1* T3801C, *CYP1A1* A2455G, and *COMT* Val158Met, were identified as susceptibility loci in breast cancer development according to recent meta-analysis reports [49,50,51]. However, these SNPs were not included in the present study because they failed in the assay design or deviated from HWE. It is worth noting that the significance of the results could be due to certain biases, which should be considered. Our study is still ongoing, and an additional large-scale assay with a more comprehensive design and more candidate SNPs may help to resolve these issues. It is, however, expected to improve the risk models for breast cancer among Han Chinese women.

## 4. Experimental Section

### 4.1. Ethics Statement

The present study was reviewed and approved by the regional ethical committee of Nanfang Hospital, Southern Medical University, Guangzhou, Guangdong Province, China (IRB No: 2009-SCBCGS-GZ-01) on 12 January 2009. Written informed consent was obtained from all of the participants in the trial.

### 4.2. Study Subjects

A total of 1076 Han Chinese women were included in the current study; 530 were cases with breast cancer and 546 were controls. All of the patients were recruited from Nanfang Hospital after a pathological diagnosis was ascertained. Clinical information was obtained from the patients’ medical records, including demographic characteristics, age at diagnosis, menopausal status, ER status, PR status, HER2 status, clinical stage, degree of differentiation, size of tumor, and lymph node involvement. The healthy controls were derived randomly and simultaneously from the same hospital, and were matched by ethnicity and geographic location. All of the controls were checked by a physician for the absence of cancer, and family history of their first-degree relatives was self-ascertained.

### 4.3. DNA Extraction

Peripheral blood samples were collected from the participants and stored at −70 °C until DNA extraction. Genomic DNA was extracted from EDTA-containing blood using the EZNA blood DNA kit (Omega Biotek, Norcross, GA, USA) according to the manufacturer’s instructions. The DNA was stored at −70 °C.

### 4.4. SNP Selection and Genotyping

The rationale for SNP selection has been described in detail elsewhere [23,52]. Briefly, we selected candidate SNPs in estrogen-related genes from Genome-Wide Association Studies GWAS, meta-analysis in multiple populations, or other large-sample association studies on breast cancer [41,44,53]. Furthermore, SNPs that were significantly associated with other hormone-induced cancers such as ovarian cancer and endometrial cancer were also selected. To achieve a power of at least 50%, with an odds ratio of 1.3, only SNPs with MAF ≥ 10% in the Chinese population according to the HapMap database were included. Monomorphic SNPs among the Chinese population were discarded. Finally, the genotyping assay was designed using Assay Design 4.0 software (Sequenom, San Diego, CA, USA), and after comprehensive evaluation, a total of 32 SNPs in 16 genes were included in this study. Details on the locations and characteristics of these SNPs are listed in Table S2.

For each SNP, a pair of amplification primers and an extension primer were designed using Assay Design 4.0 software. Genotyping was performed using the high-throughput MassARRAY iPLEX platform (Sequenom) through multiplex reactions. The genotyping rate of DNA samples was set to ≥85%.

### 4.5. Statistical Analysis

The statistical power of the case-control study was calculated using QUANTO software version 1.2.4 (University of Southern California, Los Angeles, CA, USA; http://hydra.usc.edu/gxe). We conducted a case-control study for all of the subjects, and then the patients were stratified by ER and PR status. Association analysis based on unconditional logistic regression was carried out by estimating *OR* and 95% *CI* with multiple inheritance models (codominant, dominant, recessive, overdominant, and additive) for each SNP. HWE was assessed using Fisher’s exact test and the *χ*^2^ test. The statistical tests were implemented using SPSS 13.0 software combined with the Web-based tool SNPstats (http://bioinfo.iconcologia.net/SNPstats). We also performed the AML-based global test of association for the whole estrogen-related pathway as well as for four sub-pathways after adjusting for age using software provided by the authors of Tyrer *et al.* [54]. All of the statistical analyses were two-tailed, and the significance level was set at a *p* value of 0.05.

## 5. Conclusions

In conclusion, we have presented an integrated analysis of the relationship between common variants in estrogen-related genes and breast cancer risk. Our study provides molecular genetic evidence suggesting that the independent and combined effects of these polymorphisms potentially reflect exposure to estrogen and as a consequence, an increase in breast cancer susceptibility. Our data suggest that this association could be specific to ER status. These findings may contribute to obtaining a more conclusive understanding about the underlying etiology of breast cancer and provide new strategies for breast cancer prevention, early detection, and specific treatment.

## Supplementary Materials

Supplementary materials can be found at http://www.mdpi.com/1422-0067/16/02/4121/s1.
